# Assessing the Anticholinergic Burden in the West Memory Assessment Service (MAS)

**DOI:** 10.1192/bjo.2022.460

**Published:** 2022-06-20

**Authors:** Rebecca Lees, John Alderson

**Affiliations:** Leeds and York Partnership NHS Foundation Trust, Leeds, United Kingdom

## Abstract

**Aims:**

Aim: Evaluate the recording of the Anti-Cholinergic Burden (ACB) score for patients referred to the West Leeds Memory Assessment Service (MAS). Objectives: 1) Calculate the Anti cholinergic Burden score of all patients referred to the West MAS in June 2021 where this has not already been done. 2) Determine if there is a need to review the process for assessing this component of the cognitive assessment in MAS.

**Methods:**

All patients who were referred to the West MAS in June 2021 were included in this project. Data were collected from GP referral letters, the referral meeting documentation and patients’ GP prescriptions.

These records were checked for a documented ACB score. If ACB scores were not found, they were calculated based on a patient's GP prescription. Several ACB calculators were used to do this, as NICE does not recommend a specific scoring system.

**Results:**

There were 60 referrals in June 2021. Within this data set, there were no documented ACB scores found at the point of referral.

The different scoring systems used led to considerably different ACB scores, with the lowest figure suggesting 20.4% of patients had a raised ACB score (n = 10).

In all three scoring systems used, the medication most frequently leading to a larger anticholinergic burden is Amitriptyline.

**Conclusion:**

Within the service, during the referral process we are not routinely documenting anticholinergic burden. We are in the process of agreeing a standardised ACB tool to review all new referrals to the service and determine how we can communicate these findings with referrers. We are looking to improve local awareness of ACB scoring across the memory pathway and will undertake a re-audit of practice in 3 months to establish if the proposed changes improve our results

